# From Social Attitudes to Outcomes: The Role of Institutional Quality in Development

**DOI:** 10.12688/openreseurope.20302.2

**Published:** 2026-02-14

**Authors:** Oksana Liashenko, Mª Ángeles Caraballo

**Affiliations:** 1 Economics and Trade Department, Lesya Ukrainka Volyn National University, Lutsk, 43025, Ukraine; 2 Economics and Economic History Department, University of Seville, Seville, 41018, Spain; 3 IUSEN (Instituto de Economía y Negocios de la Universidad de Sevilla), Universidad de Sevilla, Sevilla, 41018, Spain

**Keywords:** Social attitudes, institutional quality, development outcomes, gender equality, environmental protection, immigrants

## Abstract

**Background:**

Institutional quality is a critical determinant of development outcomes, yet the role of social attitudes in shaping institutions remains underexplored. This study examines the impact of public attitudes toward gender equality, environmental protection, and immigration on institutional strength and socioeconomic development.

**Method:**

Using data from Wave 7 of the World Values Survey, we apply a classification of attitudes based on a combination of set theory and ordinal preference logic. Respondents are grouped into 27 attitude combinations and then aggregated into eight categories. Country-level proportions are computed. To explore relationships among variables, we apply partial correlation network modelling using the EBICglasso algorithm with the Extended Bayesian Information Criterion (EBIC) for model selection. This network approach reveals how institutional nodes—such as the rule of law, democratic stability, and market organisation—mediate the influence of social attitudes. Composite indicators of institutional quality and development outcomes are constructed using Principal Component Analysis. We then conduct mediation analysis through path modelling with these composite scores, estimating both direct and indirect effects. Bootstrapping with 5,000 replications ensures statistical robustness.

**Results:**

Network modelling reveals that institutional quality is a key bridge between social attitudes and development outcomes. Path modelling confirms that institutional quality mediates these effects in most cases. Neutral-positive and mixed-neutral attitudes yield the most potent positive indirect effects, underscoring their role in consensus building. Negative attitudes are associated with institutional weakening and lower development performance. Interestingly, moderately negative views may drive democratic reform when linked to institutional accountability.

**Conclusion:**

Social attitudes affect development primarily through their influence on institutions. Contrary to common assumptions, moderate and neutral positions are not passive; they foster institutional adaptability and stability. These findings underscore the importance of targeting centrist groups in policy design to reinforce inclusive governance and long-term development.

## Plain Language Summary

This study examines how people’s attitudes towards gender equality, environmental protection and immigration affect the quality of institutions and, in turn, economic performance. Using global survey data, the research categorises attitudes as positive, neutral or negative and analyses their effects using advanced statistical methods.

The results show that institutions act as a bridge between social attitudes and development. Positive and neutral attitudes towards the three topics help strengthen institutions, leading to economic development. Interestingly, moderate or less extreme views (neutral attitudes) have a greater positive effect on institutional quality than positive ones. Furthermore, negative attitudes weaken institutions and hinder economic development. These findings suggest that less polarised societies are more likely to develop strong and effective institutions.

The research in this paper provides a comprehensive approach to better understanding the links between social attitudes, institutional quality and economic development. It can help policymakers design more effective and inclusive governance frameworks.

## 1. Introduction

‘Institutions Matter.’ This title from Douglass North's work ( North, 1994) highlights the influence of institutional frameworks on economic dynamics. Although institutions have long been central to economic analysis, the rise of neoclassical economics in the 20th century shifted the emphasis towards mathematical modelling, capital accumulation and technological change, relegating institutions to the background.

In recent decades, however, there has been renewed recognition of institutions' critical role in long-term development. The revival was driven by Douglass North’s Nobel Prize win in 1993, awarded ‘for having renewed research in economic history by applying economic theory and quantitative methods to explain economic and institutional change’ ( https://www.nobelprize.org/prizes/economic-sciences/1993/north/facts/).

His insights into the relationship between economic performance and institutions sparked a wave of research, further reinforced by the 2024 Nobel Prize awarded to Daron Acemoglu, Simon Johnson and James Robinson ‘for studies of how institutions are formed and affect prosperity’ ( https://www.nobelprize.org/prizes/economic-sciences/2024/press-release/). These authors propose a framework that explains the dynamic interaction between political institutions and the distribution of resources, which together shape economic institutions and outcomes (see, for instance, Acemoglu *et al.*, 2005 and Acemoglu *et al., *2012). According to their perspective, high-quality political institutions are characterised by adequate checks and balances on power and broad-based participation. These, in turn, give rise to high-quality economic institutions that encourage agents to engage in activities with greater economic returns, driving investment, human capital accumulation, technological progress and, ultimately, improved economic performance.

While this framework has faced some criticism, there is a broad consensus in the literature on the strong relationship between high-quality political and economic institutions and economic performance ( Efendic *et al.*, 2011; North, 1991 and the references therein; Acemoglu *et al.*, 2012). This raises a crucial question: What are the determinants of institutional quality? The extensive literature on this topic identifies several factors that are not mutually exclusive, such as cultural influences ( Acemoglu & Robinson, 2025; Alesina & Giuliano, 2015), legal origins ( La Porta *et al.*, 1999), various aspects of social diversity ( Alesina *et al.*, 2003; Buitrago & Caraballo, 2022; Desmet *et al.*, 2012; Desmet *et al.*, 2017) and colonial institutions ( Acemoglu *et al.*, 2001), among others.

This paper adds to the existing literature by incorporating social attitudes as a significant factor in shaping institutional quality. Social attitudes affect government structures’ responsiveness, inclusiveness and resilience, which, in turn, influence economic performance. Specifically, we focus on attitudes towards three essential and highly polarising topics in contemporary society that play a significant role in public discourse and policy-making: environmental protection, immigration and gender equality. While some evidence suggests that support for gender equality and environmental protection has strengthened institutional resilience and adaptability ( Levi & Stoker, 2000; Rodrik *et al.*, 2004), the mechanisms linking attitudes towards these critical topics to institutional quality and their subsequent impact on developmental outcomes remain largely unexplored. In an era of increasing polarisation and ideological division, understanding how these attitudes influence institutional mechanisms may be essential for fostering cohesive governance conducive to stable economic development. For example, polarised views on immigration can undermine democratic institutions by eroding trust and social cohesion ( Pevnick, 2024). Similarly, disagreements over gender equality and environmental policies can obstruct institutions from implementing inclusive and sustainable reforms.

Therefore, this paper aims to analyse how social attitudes towards the three selected topics influence institutional quality and, in turn, development outcomes. We use data from the World Values Survey (WVS) Wave 7 as a proxy for social attitudes. Methods such as set theory and ordinal preferences are used to capture the inherent complexity of these relationships. Additionally, advanced analytical tools are employed, including partial correlation network modelling estimated via the EBICglasso algorithm and selected using the Extended Bayesian Information Criterion (EBIC), implemented in JASP, alongside mediation analysis using path modelling with composite scores, to examine both the direct and mediated effects of social attitudes on development through institutional quality.

This paper makes several significant contributions. First, it integrates social attitudes into institutional quality and development framework. Second, it introduces innovative methods for operationalising these attitudes. Third, it uses advanced analytical techniques to quantify mediated relationships. Finally, the paper’s findings provide relevant information for policymakers, aiding in integrating social attitudes into sustainable governance frameworks. By bridging theoretical and methodological gaps, this study lays the groundwork for a more comprehensive understanding of the relationships between social attitudes, institutional quality and development outcomes by bridging theoretical and methodological gaps.

The remainder of the paper is structured as follows. The next section conceptualises social attitudes and explores the three selected topics. Section 3 introduces the attitude variables, classified on a scale from negative to positive using concepts from set theory and ordinal preferences, and provides an overview of the data on institutional quality and development outcomes. Section 4 examines the relationship between social attitudes, institutional quality and development outcomes, through empirical analysis using network and path modelling. Section 5 discusses the findings and their policy and governance implications, while Section 6 summarises the main conclusions and suggests areas for further research.

## 2. Social attitudes towards critical contemporary issues

**Figure 1.**
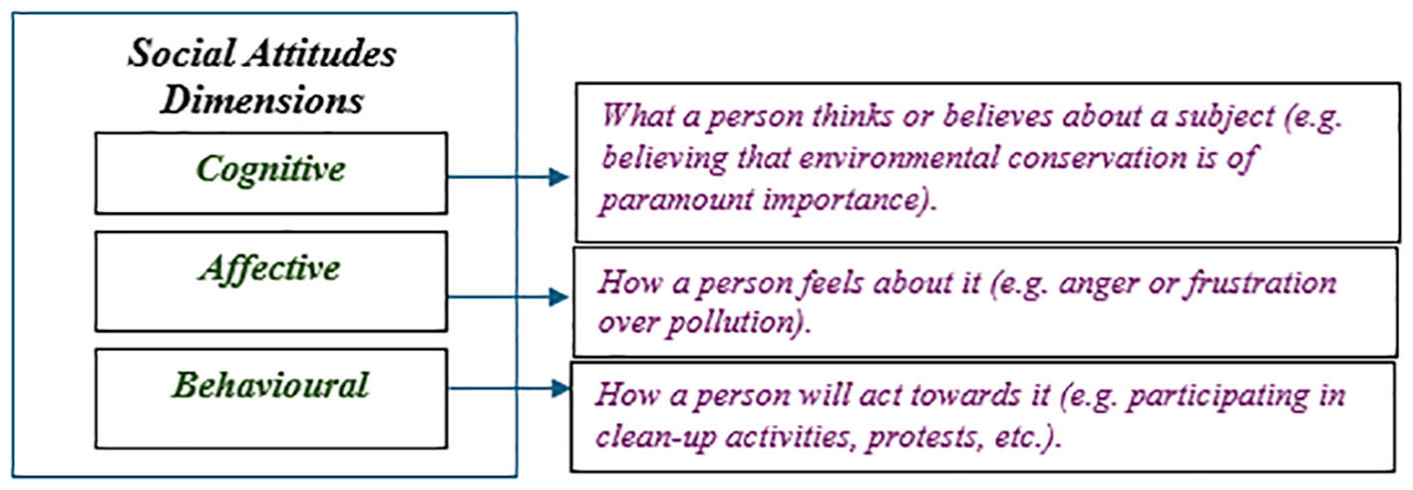
Dimensions of social attitudes.

Social Attitudes are enduring beliefs, feelings and behavioural tendencies towards socially significant objects, groups, events or symbols. They reflect individuals’ perspectives on social issues and entities, shaped by personal experiences, cultural norms, education and socialisation. As illustrated in Figure 1, these attitudes can be categorised into three dimensions. First, the cognitive dimension is concerned with beliefs about a topic. Second, the affective dimension addresses the emotions that specific subjects evoke. Finally, the behavioural dimension refers to a person's specific actions in response to those subjects ( Huajian, 2024).

This paper focuses on social attitudes towards three topics at the centre of contemporary politics. The first is the growing concern about women’s rights and gender equality. Issues such as equality in the workplace, combating gender-based violence and the demand for greater representation in leadership roles have propelled gender to the forefront of public debate, making it a key electoral issue. At the same time, growing awareness of climate change and environmental degradation has galvanised public opinion, making environmental issues a central focus for political parties and social movements. Finally, in perhaps the most developed countries, the economic and social impact of immigration has become one of the most polarising features of public debate, leading to the rise of populist and far-right political parties.

Attitudes towards these three topics and institutional quality are closely related, as diverse social attitudes can influence how institutions function and prioritise these issues. At the same time, institutions can shape and promote certain attitudes. For instance, positive attitudes towards gender equality may lead to greater representation of women in political and decision-making roles, which is positively linked to institutional quality (see Hessami & Lopes da Fonseca, 2020 for a review of the literature supporting this evidence). At the same time, there is a feedback loop, as high-quality institutions encourage conditions conducive to gender equality.

Furthermore, societies with robust pro-environmental attitudes may encourage better environmental practices ( Drews & van den Bergh, 2016), increasing the likelihood that institutions will act in the public interest. Similarly, citizens are more inclined to support and engage in sustainable practices when the institutional framework ensures the effectiveness of those practices ( Holum & Jakobsen, 2024). In the same way, high-quality institutions foster greater trust among citizens in institutions themselves, enhancing overall social cohesion and tolerance. In such environments, immigrants are often seen as contributing to society rather than competing for scarce resources, leading to more favourable attitudes towards immigration ( Halapuu *et al.*, 2014). It is also important to note that positive attitudes towards immigrants are a feature of tolerant societies. As highlighted by Buitrago *et al.* (2019), tolerance is essential for achieving social cohesion in diverse societies, as it facilitates the integration of all community members. This helps to overcome tensions caused by diversity and leads to more stable governance.

Three questions from Wave 7 of the WVS were selected to analyse attitudes towards these topics ( Table 1). These questions pertain to the cognitive dimension of social attitudes, as outlined in Figure 1. Positive attitudes support gender equality and environmental protection, and view immigration as beneficial for development. In contrast, neutral attitudes are indifferent to these issues and negative attitudes are explicitly opposed to them.

**Table 1.** Selected questions.

	Gender equality	Environment	Immigration
***Attitude***			
	**Q33 Jobs scarce: Men should have more right to a job than women**	**Q111 Protecting environment vs. Economic growth**	**Q121 Impact of immigrants on the development of the country**
***Positive***	4. Disagree 5. Disagree strongly	1. Protecting environment	4. Quite good 5. Very good
***Neutral***	3. Neither agree nor disagree	3. Other answer	3. Neither good, nor bad
***Negative***	1. Agree strongly 2. Agree	2. Economic growth	1. Rather bad 2. Quite bad

For gender equality, we selected Q33 from nine possible statements, preceded by the following questions: *How would you feel about the following statements? Do you agree or disagree with them?* Q33 reads: *When jobs are scarce, Men should have more right to a job than women.* The respondents can choose between *Agree strongly*, *Agree*, *Neither agree nor disagree*, *Disagree*, *Disagree strongly.* This question captures anti-egalitarian and discriminatory attitudes towards women in the labour market, offering insight into an individual’s views on women's social and economic roles. It has been used in studies linking gender attitudes to various socioeconomic outcomes, such as progress in gender-sensitive policies, the quality of employment and women’s participation in the workforce (see, for instance, Fortin, 2005; Leschke & Jepsen, 2014; Zawaira *et al.*, 2023).

The question selected for attitudes towards the environment is Q111: *Here are two statements people sometimes make when discussing the environment and economic growth. Which of them comes closer to your own point of view?* The possible answers are: *Protecting the environment should be given priority, even if it causes slower economic growth and some loss of jobs; economic growth and creating jobs should be the top priority, even if the environment suffers to some extent* and *other answer.* It reflects the respondent’s willingness to prioritise long-term environmental sustainability over immediate economic gains, indicating their level of social commitment to ecological issues. This question has been widely used in studies analysing attitudes towards environmental protection ( Li *et al.*, 2018; Mondéjar-Jiménez *et al.*, 2012) and in research linking these attitudes to topics such as the design of environmental policies ( Holum & Jakobsen, 2024), the achievement of Sustainable Development Goals ( Liashenko *et al.*, 2026) and political polarisation ( Birch, 2020).

Finally, attitudes towards immigration were analysed using question Q121: *Now we would like to know your opinion about the people from other countries who come to live in [your country] - the immigrants. How would you evaluate the impact of these people on the development of [your country]?* Respondents are asked to choose between *Very good*, *Quite good*, *Neither good nor bad*, *Quite bad* and *Very bad.* It is a measure of respondents’ views on the potential burden of immigration on a country’s resources. This question serves as a proxy for social attitudes towards immigration by gauging whether individuals perceive immigrants as beneficial or burdensome to the national economy. Such perceptions are crucial for understanding social acceptance or resistance to immigration within a society. This question, or similar ones in other surveys, has been used in research on the determinants and consequences of attitudes towards immigrants ( Chang & Kang, 2018; Halapuu *et al.*, 2014; Kaeser & Tani, 2023).

A brief summary of the responses to these questions provides an initial overview of global social attitudes towards the three selected topics. The sample includes 59 countries from all major geographical regions worldwide. The data show that a substantial proportion of respondents disagree (30.19%) or strongly disagree (14.78%) with the statement presented in Q33. However, a notable percentage of respondents agree (22.58%) or strongly agree (17.10%), indicating a sizeable portion that is not in favour of gender equality. The neutral responses (14.62%) suggest that some individuals are undecided or indifferent. Furthermore, more than half of respondents (54.32%) believe that protecting the environment should take priority, even at the cost of slower economic growth and some job losses. This reflects a strong positive attitude towards environmental protection. A small percentage of respondents (2.90%) selected ‘other’, suggesting neutrality or alternative views not captured by the main response categories. Many respondents (38.32%) believe that economic growth and job creation should take priority, even at the expense of the environment. This highlights a substantial proportion of the population that prioritises economic growth over environmental concerns. Finally, 28.23% of respondents believe that immigrants have a positive impact on their country’s development, with 21.64% considering this to be quite good and 6.58% very good. The largest segment, 39.47%, has a neutral attitude, perceiving the impact of immigrants as neither good nor bad. A total of 29.79% of respondents have a negative view of the impact of immigrants, with 19.25% considering it to be quite bad and 10.54% considering it to be rather bad.

## 3. Data and variables

### 3.1. Measuring country-level variability in social attitudes

Organising and grouping similar responses is essential for reducing dimensionality, as it helps to identify patterns within the data (see Table 2). Structuring responses into coherent groups of attitudes based on the co-occurrence of response variants clarifies the relationships between variables, simplifies the interpretation of results and enables meaningful conclusions to be drawn from the WVS datasets. By combining the responses to the three selected questions, we categorise attitudes on a spectrum from negative to positive.

**Table 2.** Attitude groups based on co-occurrence of response variants.

*Q33*	*Q111*	*Q121*	*Attitude groups*
**Negative (-)** (Agree strongly, Agree)	Economic growth (-)	Neither good, nor bad (n/n)	At1
		Bad (-)	At2
		Good (+)	At3
	Other answer (n/n)	Neither good, nor bad (n/n)	At4
		Bad (-)	At5
		Good (+)	At6
	Protecting environment (+)	Neither good, nor bad (n/n)	At7
		Bad (-)	At8
		Good (+)	At9
**Positive (+)** (Disagree strongly, Disagree)	Economic growth (-)	Neither good, nor bad (n/n)	At10
		Bad (-)	At11
		Good (+)	At12
	Other answer (n/n)	Neither good, nor bad (n/n)	At13
		Bad (-)	At14
		Good (+)	At15
	Protecting environment (+)	Neither good, nor bad (n/n)	At16
		Bad (-)	At17
		Good (+)	At18
**Neutral (n/n)** (Neither agree nor disagree)	Economic growth (-)	Neither good, nor bad (n/n)	At19
		Bad (-)	At20
		Good (+)	At21
	Other answer (n/n)	Neither good, nor bad (n/n)	At22
		Bad (-)	At23
		Good (+)	At24
	Protecting environment (+)	Neither good, nor bad (n/n)	At25
		Bad (-)	At26
		Good (+)	At27

To make the attitude groups presented in Table 2 more coherent, we use the following logical structure, which clusters attitudes into positive, neutral and negative groups:

**1. Positive attitudes:**

- *Strongly positive* ( *Pos_s*): this group includes individuals who are strongly supportive of gender equality and environmental protection and view immigration as having a positive impact on economic performance.

- *Moderately positive (Pos_m)*: individuals in this group exhibit moderately positive attitudes. These respondents may still show support for the three selected statements but their support is less intense than that of the strongly positive group.

**2. Neutral attitudes (various degrees of neutrality):**

- *Neutral positive (Neu_p)*: individuals in this group tend to be slightly positive but are generally neutral.

- *Strongly neutral* ( *Neu_s*): this group comprises individuals who consistently maintain a neutral stance.

- *Neutral mixed (Neu_m)*: this group brings together individuals who hold a combination of positive, negative and neutral stances.

- *Neutral negative (Neu_n)*: respondents in this group are generally neutral but are slightly inclined towards a negative stance.

**3. Negative attitudes:**

- *Strongly negative* ( *Neg_s*): this group consists of individuals who most strongly disagree with the three specific topics. They are likely to favour men when jobs are scarce or prioritise economic growth over environmental protection.

- *Moderately negative* ( *Neg_m*): this group includes individuals with moderately negative responses. While they may still hold negative views, their opinions are less intense than those of the strongly negative group.

Each of the eight attitude groups yields a variable (see Table 3) that will be used in further empirical analysis. Each variable is calculated as the proportion of individuals exhibiting the same response pattern relative to the total number of respondents in a given country covered by WVS Wave 7.

**Table 3.** Aggregated groups and variables.

*Level*	*Attitude groups*	*Variable*
*Positive*		
Strongly (+,+,+)	At18	*Pos_s*
Moderately (-,+,+)(n/n,+,+)	At12, At15, At16, At17, At9, At27	*Pos_m*
*Neutral*		
Neutral positive (n/n,n/n,+)	At24, At13, At25	*Neu_p*
Strongly neutral (n/n,n/n,n/n)	At22	*Neu_s*
Neutral mixed (n/n,-,+;)	At26, At21, At14, At10, At7, At6	*Neu_m*
Neutral negative (n/n,n/n,-)	At19, At4, At23	*Neu_n*
*Negative*		
Moderately (-,-,+)(-,-,n/n)	At1, At3, At20, At5, At8, At11	*Neg_m*
Strongly (-,-,-)	At2	*Neg_s*

Note: The attitude displayed towards Q33, Q111 and Q121, respectively, is indicated in parentheses, based on the classifications in Table 2. For example, (n/n, -, +) means that, according to Table 2, individuals responded ‘Neither agree nor disagree’ to Q33, ‘Economic growth and jobs creation’ to Q111 and ‘Good’ to Q121.

The grouping of attitudes draws on concepts from set theory and ordinal preferences. While we do not directly apply social choice theorems, our approach follows the methodological tradition in economics and sociology of working with ordinal rankings without imposing arbitrary numerical scales. Arrow’s work on social choice theory uses ordinal preferences to explain how individual rankings can be aggregated into a collective decision ( Arrow, 1951). His Impossibility Theorem illustrates the challenges in creating a social welfare function that reasonably combines individual ordinal preferences, underscoring the importance of the ordinal approach in understanding group preferences. Similarly, the Median Voter Theorem assumes that voters have single-peaked ordinal preferences. Black (1948) demonstrates that in a majority-rule voting system, the median voter’s preferences will prevail, emphasising the role of ordinal rankings in predicting collective decisions. This theorem demonstrates how individual preferences can be combined to reflect social decisions without the need for precise utility measurements. In the context of public goods, Bergstrom and Goodman (1973) use ordinal preferences to aggregate individual valuations, enabling the analysis of collective demand without relying on cardinal utility measurements. From a sociological perspective, Alwin and Krosnick (1985) show that ordinal rankings provide a reliable method for measuring attitudes without assigning numerical values.

Thus, set theory and ordinal preferences provide a structured way to organise respondents’ attitudes into coherent groups without imposing arbitrary numerical scales. This approach preserves the inherent order of preferences while capturing the nuances of social attitudes. In set theory, each attitude group ( *At1 to At27*) is treated as a set of respondents who share certain combinations of survey responses. Defining these sets allows us to use set operations to merge them into broader categories based on common characteristics. For example, the moderately negative attitude group ( *Neg_m*) is formed by combining the sets *At1, At3, At5, At8, At11* and *At20.* This approach helps to organise respondents into aggregated *Negative, Positive* and *Neutral* groups based on their shared responses.

The use of ordinal preferences enables us to rank respondents’ attitudes without assigning numerical values to the differences between them. This means that we can order the attitudes from strongly negative to strongly positive (e.g. *Neg_s ≺ Neg_m ≺ Neu_n ≺ Neu_m ≺ Neu_s ≺ Neu_p ≺ Pos_m ≺ Pos_s*) based on the level of agreement or disagreement. We then aggregate the groups according to this ordinal ranking, preserving the relative order of preferences. This method respects the inherent ranking in respondents’ attitudes without making assumptions about the exact size of the differences between preference levels.

A preliminary analysis of the data (Table A.I.1 in Appendix A.I)(Extended data) provides an initial overview of the social attitude patterns based on the categorisation described above. To obtain an overall perspective, we classify countries into broad geographical regions: North America, Latin America and the Caribbean, Europe, Africa, the Middle East and North Africa (MENA), Asia-Pacific and the Post-Soviet states (including Central Asia). Strongly positive and moderately positive attitudes are prevalent in North America and Western Europe. In contrast, Africa, MENA and parts of Asia-Pacific and the Post-Soviet states exhibit a higher prevalence of moderately and strongly negative attitudes. A mixed pattern emerges in Latin America and Asia-Pacific, where moderately positive attitudes dominate but are tempered by significant neutral or negative attitudes. This heterogeneous landscape may have varying implications for institutional quality and development outcomes, as discussed in Section 4.

### 3.2. Institutional Quality and Development Outcomes

**Figure 2.**
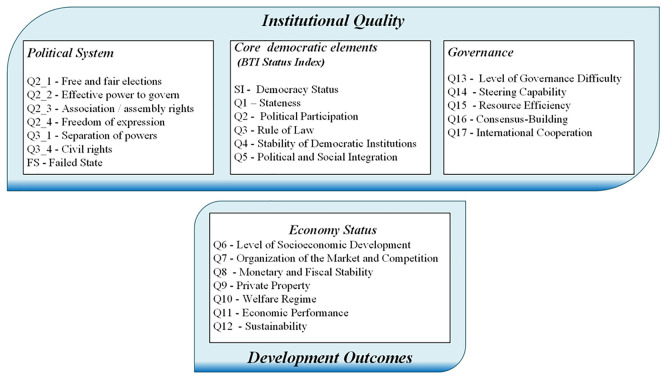
Institutional Quality and Development Outcomes Framework ( BTI. Bertelsmann Stiftung’s Transformation Index).

As outlined in the Introduction, this paper analyses the relationships between social attitudes, institutional quality and development outcomes. As a proxy for the latter two variables, we use data from the Institutional Quality and Development Outcomes Framework derived from the Bertelsmann Stiftung’s Transformation Index (BTI) ( Figure 2). This database defines three dimensions of institutional quality. The first, the *Political System*, focuses on operational rights within the democracy, including free elections, civil liberties and checks on governmental power, with particular attention to civil rights and the risk of state failure. The level of democracy is determined using the *Status Index,* which evaluates core aspects such as state authority, political participation, the rule of law and the stability of democratic structures. This index serves as a baseline for democratic integrity and social cohesion. The third dimension of institutional quality, *Governance*, encompasses indicators that measure government efficiency, resource management, policy implementation and the capacity for consensus building and international cooperation. These factors reflect the quality of administrative functions essential for stability and growth.

These variables serve as indicators of the quality of political institutions, in line with the framework proposed by Acemoglu *et al.* ( 2005; 2012). As discussed in the Introduction, their work highlights the dynamic relationship between political institutions and resource distribution, which together shape economic institutions and outcomes. These two elements can be analysed using the Development Outcomes indicators, which include data on the quality of economic institutions, such as Private Property and the Organisation of the Market and Competition, as discussed by Acemoglu *et al.* ( 2001; 2005; 2012). However, these indicators also incorporate variables that measure socioeconomic outcomes, such as Economic Performance and Level of Socioeconomic Development. ^1^

## 4. A multidimensional framework linking social attitudes, institutional quality and development outcomes

This section examines the potential relationship between social attitudes towards the three selected topics (environmental protection, gender equality and immigration), institutional quality and economic outcomes. To this end, we first employ network-based modelling of conditional dependencies using a data-driven approach. This exploratory network analysis helps identify central and highly connected institutional indicators within the system. Building on these results, we then assess the direct and mediated effects of social attitudes on development outcomes using mediation analysis through path modelling with composite scores.

### 4.1. Network Analysis of variable relationships and pathways

Network analysis provides an exploratory framework for identifying conditional dependencies among variables, making it a suitable first step before conducting mediation analysis using path modelling.

To explore the structure of relationships among social attitudes, institutional quality, and development outcomes, we employ network analysis based on partial correlation structures. This approach allows us to identify conditional dependencies among variables in a data-driven manner, thereby highlighting central, highly connected nodes within the system. Rather than imposing a predefined causal structure, the analysis serves an exploratory role, supporting the formulation of hypotheses about how social attitudes and institutional features are interrelated.

Network structures are estimated using the EBICglasso estimator. This method applies L1-regularisation to the inverse covariance matrix and selects the optimal network structure using the Extended Bayesian Information Criterion (EBIC). The resulting networks are interpreted as partial correlation structures rather than as classic causal Bayesian graphs. In this context, the term “Bayesian” refers exclusively to the information criterion used for model selection within the EBICglasso algorithm, rather than to Bayesian probabilistic network modelling. This terminology follows the JASP Network module documentation.

The resulting network representation provides a data-driven map of associations among institutional and development indicators, which informs the subsequent mediation analysis using path modelling with composite scores. While the network itself does not establish causal relationships, it offers an empirically grounded basis for specifying and interpreting mediated pathways in the next stage of the analysis, thereby enhancing model coherence and interpretability ( Lauritzen, 1996).

It is important to note that the estimated networks do not provide probabilistic measures of association. Instead, edges represent regularised partial correlation coefficients, indicating the presence and relative strength of conditional dependencies among variables.

***Network Analysis for development outcome and institutional quality variables.*** In this step, we first perform a NA focusing on development outcome variables, followed by a second NA incorporating institutional quality variables. A comparison of these networks reveals key relationships in development outcomes and assesses the impact of institutional quality, shedding light on development pathways and policy opportunities.

The Development Outcomes Network comprises seven nodes with ten non-zero edges out of a possible 21, yielding a sparsity of 0.524 (Table A.I.2. in Appendix A.I) (Extended data). This moderate sparsity suggests a limited network structure, where specific development outcomes are selectively connected. Rather than exhibiting widespread interconnectivity, this network highlights targeted relationships between variables. The combined Institutional Quality and Development Outcomes Network consists of 25 nodes with 70 non-zero edges out of a possible 300, resulting in a sparsity of 0.767 (Table A.I.4. in Appendix A.I) (Extended data). This higher sparsity reflects a more selective network structure, where only the most essential connections between institutional and developmental indicators are retained. This emphasises critical pathways while maintaining simplicity.

In the Development Outcomes Network, Organisation of the Market and Competition is strongly connected to variables related to fiscal stability, private property and welfare regimes, underlining the central role of market structure in strengthening governance and economic resilience. Similarly, the combined network shows that the Rule of Law is closely linked to democratic stability and consensus-building, emphasising the importance of legal frameworks and cooperative mechanisms in fostering governance resilience.

Centrality measures (Figures A.I.1 and A.I.2, Tables A.I.3 and A.I.5 in Appendix A.I) (Extended data) reveal key variables in both networks. In the Development Outcomes Network, market-related factors and welfare regimes emerge as central connectors that link institutional elements to development outcomes. In the combined network, the Rule of Law bridges institutional stability and broader development metrics. Meanwhile, sustainability and governance factors ensure continued connectivity and responsiveness within the network. Influential nodes in the combined network include political participation and the Rule of Law, while market-related variables are central to the Development Outcomes Network.

Both networks show robust associations between variables. The Development Outcomes Network highlights strong connections between market structure, fiscal stability, private property and welfare regimes. In the combined network, significant links between political participation, governance effectiveness and democratic stability validate the robustness of these relationships.

***Network Analysis of attitudes and BTI variables.*** To better understand the impact of social attitudes on institutional and development variables ( Figure 2) we conducted two NAs: one focusing on attitudes and development outcome indicators (DOA network) and another incorporating attitudes and institutional quality indicators (IQA network). The DOA network contains 15 nodes with 41 non-zero edges out of a possible 105, yielding a sparsity of 0.610 (Table А.І.6 in Appendix A.I) (Extended data). This moderate sparsity suggests that the network captures key connections between development indicators and social attitudes. Meanwhile, the IQA network consists of 25 nodes and 90 non-zero edges out of a possible 300, resulting in a sparsity of 0.700 (Table А.І.8 in Appendix A.I) (Extended data). This higher sparsity reflects a more selective network structure, emphasising critical dependencies while minimising peripheral connections.

These differences in network density reflect a comparative focus: the DOA network connects a broader range of development-related indicators, while the IQA network prioritises core relationships that reinforce governance and democratic stability. Centrality metrics (Figures A.I.3 and A.I.4, Tables A.I.7 and A.I.9 in Appendix A.I) (Extended data) highlight key nodes that serve as significant connectors or hubs within each network. These influential variables play a crucial role in bridging or shaping other indicators. Table 4 provides a summary of the hub variables identified through NA.

**Table 4.** Key hub variables and their roles in the DOA and IQA networks.

Network	Hub Variables	Role/Significance
**DOA Network**	*Q7*	Central to linking market structure with financial stability, property rights and sustainability outcomes.
	*Q10*	Acts as a bridge between welfare policies and sustainability, reflecting their systemic role in development.
	*Neg_m*	Acts as a bridge between moderately negative social attitudes and market dynamics and welfare reforms.
	*Pos_s*	Reinforces welfare and sustainability, reflecting the constructive role of positive social attitudes.
**IQA Network**	*Q3*	Key foundation for institutional resilience, linking legal frameworks with governance and democratic stability.
	*Q4*	Anchors institutional quality by connecting governance stability with public attitudes and civic rights.
	*Neg_m*	Links moderately negative social attitudes to civic rights and democratic reforms, emphasising the potential for positive change.
	*Pos_s*	Strengthens governance quality, indirectly supporting institutional performance and stability.

*Q3*: Rule of Law; *Q4*: Stability of Democratic Institutions; *Q7*: Organisation of the Market and Competition; *Q10*: Welfare Regime; *Neg_m*: Neutral Mixed; *Pos_s*: Strongly Positive.

Furthermore, the NA within the DOA and IQA networks reveals the relationships between social attitudes, institutional quality and development outcomes, as shown in Table 5. These results provide a comprehensive understanding of how social attitudes shape institutional quality and development through distinct mechanisms, providing valuable insights for policy interventions.

**Table 5.** Key pathways identified in the DOA and IQA networks.

Network	Pathway	Description
**DOA Network**	*Q7→Q9→Q12*	Market organisation affects property rights, which in turn drives sustainability outcomes.
	*Q10 → Q12*	Welfare policies are directly linked to sustainability, reflecting the role of social systems in development.
	*Neg_m→ Q7→Q10*	Moderately negative social attitudes impact market structure, which influences welfare provisions and policy reforms.
**IQA Network**	*Q3 → Q4*	Legal frameworks directly reinforce democratic stability, forming the basis of governance resilience.
	*Q2 → Q2_4*	Civic participation strengthens the effectiveness of governance, highlighting the role of engagement in institutional performance.
	*Neg_m → Q4*	Moderately negative social attitudes drive reforms aimed at democratic stability and governance improvements.
	*Pos_s → Q13*	Positive attitudes reinforce governance quality, indirectly enhancing institutional resilience.

*Q2*: Political Participation; *Q2_4*: Freedom of Expression; *Q3*: Rule of Law; *Q4*: Stability of Democratic Institutions; *Q7*: Organisation of the Market and Competition; *Q9*: Private Property; *Q10*: Welfare Regime; *Q12*: Sustainability; *Q13*: Level of Governance Difficulty; *Neg_m*: Neutral Mixed; *Pos_s*: Strongly Positive.

Finally, the results of the DOA and IQA networks, along with their policy implications, are summarised and compared in Table 6.

**Table 6.** Comparison of the roles, hubs and pathways across the DOA and IQA networks.

Aspect	DOA Network	IQA Network
**Key Hubs**	*Q7 , Q10, Neg_m, Pos_s*	*Q3, Q4 , Neg_m, Pos_s*
**Primary Focus**	Linking attitudes to economic development outcomes.	Connecting governance quality, legal frameworks and social attitudes to institutional resilience.
**Pathways**	Economic policies: *Q7 → Q9 →Q12*	Governance reforms: Q3 → Q4
	Welfare reforms: *Q10 → Q12*	Civic engagement: Q2 → Q2_4
	Moderately negative attitudes loop: *Neg_m→Q7→ Q10*	Moderately negative attitudes loop: *Neg_m* → Q4
**Policy Implications**	Focus on market stabilisation, addressing moderately negative attitudes and promoting welfare and sustainability.	Strengthening legal frameworks and democratic stability while leveraging positive social attitudes.

*Q2*: Political Participation; *Q2_4*: Freedom of Expression; *Q3*: Rule of Law; *Q4*: Stability of Democratic Institutions; *Q7*: Organisation of the Market and Competition; *Q9*: Private Property; Q *10*: Welfare Regime; *Q12*: Sustainability; *Neg_m*: Neutral Mixed; *Pos_s*: Strongly Positive.

### 4.2. Mediation Analysis Using Path Modelling. The mediation role of institutional quality

The NA conducted in Section 4.1 identifies relationships between social attitudes, institutional quality and development outcomes, providing a valuable initial perspective. However, further in-depth analysis is needed to better understand and quantify these relationships with greater precision.

**Figure 3.**
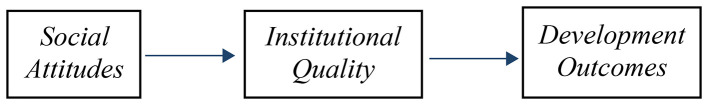
Model concept and hypothesis.

As discussed in Section 3.1, we hypothesise that political institutions, represented by the Institutional Quality variables in Figure 2, shape economic institutions and outcomes, which are measured by the Development Outcomes indicators in Figure 2. Within this framework our hypothesis is that social attitudes influence institutional quality, i.e. we propose that social attitudes influence development outcomes through their effect on institutions ( Figure 3). Given the sample size constraints (n=59 countries), we employ path analysis with composite scores rather than full latent variable structural equation modelling. Recent methodological literature supports this approach for small samples: Yuan *et al.* (2024) demonstrate that path analysis with weighted composites yields greater signal-to-noise ratios than SEM for mediation analysis, while Kelcey (2019) shows superior small-sample properties, and Savalei (2019) confirms that such methods produce estimates comparable to full SEM while being more robust to model misspecifications. Path modelling with bootstrap inference is used to test this hypothesis. This methodology allows for the creation of a latent variable representing institutional quality and assesses the impact of social attitudes on development outcomes both directly and indirectly, with institutional quality acting as a mediator.

The use of PCA to construct composite indicators is well established in the methodological literature on multidimensional measurement. Mazziotta and Pareto (2013) demonstrate that PCA is particularly appropriate when the objective is to synthesise related indicators into summary measures that preserve as much information as possible from the original data. Furthermore, Mazziotta and Pareto (2018) establish clear validity criteria for PCA-based composites: indicators should be conceptually related, the first component(s) should explain a substantial proportion of variance, and loadings should be relatively balanced across indicators. Following the analytical framework adopted in this study, we construct composite measures for the mediator and outcome nodes shown in Figure 3. Principal Component Analysis (PCA) was conducted to derive composite indicators for Institutional Quality and Development Outcomes (Appendix A.II) (Extended data). Table 7 shows the loadings for the first three principal components ( *PC1_iq*, *PC2_iq*, *PC3_iq*) in relation to Institutional Quality variables. Based on eigenvalues and explained variance, *PC1_iq* and *PC2_iq* were selected, accounting for 89% of the total variance (Table A.II.1in Appendix A.II.) (Extended data). The scree plot supports this choice, displaying an ‘elbow’ after *PC2_iq* (Figure A.II.1. in Appendix A.II.) (Extended data), confirming the inclusion of *PC1_iq* and *PC2_iq* in the mediation analysis. Similarly, Table 7 displays the principal components for Development Outcomes ( *PC1_out*, *PC2_out*, *PC3_out*). In this case, only *PC1_out* is selected for the mediation analysis, as it accounts for 83% of the variance (Table A.II.2 in Appendix A.II.) (Extended data), exceeding the Kaiser criterion threshold (Figure A.II.2 in Appendix A.II.) (Extended data). Although *PC2_out* marginally meets the threshold, it provides minimal additional explanatory power. The high proportion of variance explained by the retained components (89% for Institutional Quality; 83% for Development Outcomes) exceeds conventional thresholds recommended in the composite indicators literature ( Mazziotta & Pareto, 2013). Moreover, the balanced loading structures in Table 7 confirm that our composites adequately represent the constituent domains without being dominated by any single indicator, satisfying the validity criteria outlined by Mazziotta and Pareto (2018).

**Table 7.** Loadings of Principal Components.

**Institutional Quality Variables**					**Development Outcomes variables**			
**Variable**	*PC1_iq*	*PC2_iq*	*PC3_iq*		**Variable**	*PC1_out*	*PC2_out*	*PC3_out*
*Q1*	0.20	0.41	0.32		*Q6*	0.36	0.56	0.37
*Q2*	0.26	-0.22	0.10		*Q7*	0.40	-0.14	-0.34
*Q3*	0.27	-0.04	-0.03		*Q8*	0.37	-0.47	0.09
*Q4*	0.26	-0.16	0.09		*Q9*	0.38	-0.16	-0.58
*Q5*	0.25	-0.14	0.01		*Q10*	0.39	0.34	-0.12
*Q13*	-0.21	-0.32	-0.39		*Q11*	0.36	-0.45	0.62
*Q14*	0.23	0.29	-0.40		*Q12*	0.39	0.31	0.03
*Q15*	0.23	0.30	-0.32					
*Q16*	0.26	-0.05	-0.20					
*Q17*	0.24	0.15	-0.51					
*Q2_1*	0.25	-0.20	0.16					
*Q2_2*	0.25	-0.18	0.22					
*Q2_3*	0.25	-0.23	0.03					
*Q2_4*	0.24	-0.24	-0.05					
*Q3_1*	0.25	-0.17	0.03					
*Q3_4*	0.27	-0.01	-0.03					
*FS*	0.19	0.48	0.29					

The explanatory variable, Social Attitudes, is denoted as *X _a_* ∈ *R* where *a* ∈ { *Pos_s, Pos_m, Neu_s, Neu_p, Neu_m, Neu_n, Neg_s, Neg_m*}.

Given that our explanatory variables represent distinct but interrelated aspects of a broader construct (Tables A.III.1, A.III.2 and A.III.3 in Appendix A.III) (Extended data), we perform separate mediation analyses for each attitude to mitigate existing multicollinearity issues. This approach ensures that the effect of each explanatory variable on *PC1_out* is accurately estimated without distortion caused by high correlations between the explanatory variables (Table A.III.1 in Appendix A.III) (Extended data).

All coefficients required for the mediation analysis are explicitly estimated and reported, including the paths from social attitudes to institutional quality (X→M), from institutional quality to development outcomes (M→Y), the direct effects (X→Y), and the indirect and total effects. Inference is based on bootstrapped standard errors and confidence intervals, following standard practice in regression-based mediation analysis.

Therefore, for each attitude variable *X __a__* we estimate the following three-equation mediation model:

1. Effect of social attitudes on mediators ( *PC1_ iq* and *PC2_iq*):


$$PC1\_iq=\alpha_{1,a}X_{a}+\varepsilon_{1}$$ (1)

$$PC2\_iq=\alpha_{2,a}X_{a}+\varepsilon_{2}$$ (2)

2. Effect of mediators on development outcomes ( *PC1_out*) and direct effect of social attitudes:


$$PC1\_out=\beta_{1}PC1\_iq+\beta_{2}PC2\_iq+\delta_{a}X_{a}+\varepsilon_{3}$$ (3)

In these equations, *α _1,a_* and *α _2,a_* capture the effect of social attitudes on institutional quality. *β _1_* and *β _2_* estimate the effect of the mediators on the outcome and *δ _a_* represents the direct effect of each social attitude on *PC1_out* after accounting for mediation. Therefore, the indirect effect of each social attitude on *PC1_out* is *α _1,a_ β _1_ + α _2,a_* *β _2_*, where the first and second addends are the indirect effect via *PC1_iq* and *PC2_iq*, respectively.

As this analysis employs path modelling with composite scores rather than latent variable SEM with measurement models, traditional fit indices (CFI, TLI, RMSEA, SRMR) are not applicable to our model specification. Our just-identified mediation models (degrees of freedom = 0) are assessed through bootstrapped confidence intervals for indirect effects and explained variance for each equation, which are standard criteria for regression-based mediation analysis ( Hayes, 2017).

The mediation analysis is conducted using regression-based mediation, estimated through maximum likelihood estimation and bootstrapping for robust inference. Bootstrapped standard errors (5,000 repetitions) were computed to assess mediation and to generate robust confidence intervals for total, direct and indirect effects. In addition, the significance of indirect and total effects was evaluated using a nonlinear combination of coefficients, allowing for statistical significance testing and the construction of 95% confidence intervals ( Davison & Hinkley, 1997; Hayes, 2017).

Table 8 shows the estimated direct, indirect and total effects of each social attitude on development outcomes.

**Table 8.** Mediation analysis.

Social attitude	Total Effect	Direct Effect	Indirect Effect	Effect via *PC1_iq*	Effect via *PC2_iq*
*Pos_s*	12.55 ** (0.043)	1.85 (0.556)	10.70 ** (0.043)	8.18 (0.179)	2.52 (0.368)
*Pos_m*	4.04 * (0.074)	2.19 ** (0.039)	1.85 ** (0.014)	1.85 ** (0.014)	-0.00 (—)
*Neu_p*	35.14 *** (0.002)	7.10 (0.172)	28.04 *** (0.002)	23.27 ** (0.013)	4.77 ** (0.032)
*Neu_s*	79.74 (0.507)	-24.03 (0.689)	103.77 ** (0.025)	-25.87 (0.818)	129.64 ** (0.025)
*Neu_m*	25.65 ** (0.048)	0.16 (0.973)	25.49 ** (0.048)	20.80 ** ( *0.038*)	4,69 ** (0.027)
*Neu_n*	17.45 ** (0.049)	-2.11 (0.352)	19.56 ** (0.049)	10.41 ** (0.042)	9.15 ** (0.015)
*Neg_m*	-11.42 *** (0.001)	-2.35 (0.123)	-9.08 *** (0.001)	-7.39 *** (0.001)	-1.69 (0.332)
*Neg_s*	-10.33 *** (0.003)	-1.42 (0.347)	-8.91 *** (0.003)	-7.80 *** (0.003)	-1.11 (0.266)

*Note:* p-values in parenthesis *<0.10, **p<0.05 and *** p<0.01

As shown in Table 8, the total effect indicates that social attitudes (except for *Neu_s*) influence development outcomes. Negative attitudes (both *Neg_m* and *Neg_s*) have a negative effect, whereas all other attitudes contribute positively. However, when the total effect is decomposed, it becomes evident that the indirect effect is the main driver for this relationship, as the direct effect is significant only for *Pos_m.* In contrast, the indirect effect is significant for all attitudes and remains negative for negative attitudes. For neutral attitudes (except for *Neu_s*), mediation is complete, meaning that the indirect effect operates through both institutional variables ( *PC1_iq* and *PC2_iq*). For the remaining attitudes, mediation is partial, occurring through only one of the institutional variables. It is interesting to note that neutral attitudes exert a stronger influence than positive ones.

The findings for *Pos_s* are particularly noteworthy, as the indirect effect is significant despite neither of the individual indirect effects through *PC1_iq* or *PC2_iq* reaching statistical significance. This is a well-known statistical phenomenon in which multiple minor effects, when aggregated, can lead to considerable overall mediation ( Hayes, 2017). Additionally, significance testing of individual pathways often has limited statistical power, particularly in smaller samples ( Davison & Hinkley, 1997). We also used bootstrap estimation, which offers a more robust assessment of indirect effects. This approach confirms that the total indirect effect is statistically significant ( Davison & Hinkley, 1997), even though individual pathways do not reach conventional significance levels. We can therefore conclude that *Pos_s* positively influences *PC1_out* through institutional variables.

Finally, the effects of institutional quality on development outcomes remain consistent across all models. Both *PC1_iq* and *PC2_iq* have a statistically significant impact on *PC1_out*, indicating a robust relationship regardless of model specification ( Table 9).

**Table 9.** Effects of institutional quality on development outcomes.

*β _1_*	0.54 (0.001) ***
*β _2_*	0.68 (0.001) ***

*Note:* p-values in parenthesis *** p<0.01

## 5. Discussion and implications

This study explores how social attitudes towards gender equality, environmental protection and immigration influence institutional quality and development outcomes, using a novel mixed-methods approach that combines Network Analysis (NA) and regression-based mediation analysis. Our results show that institutional quality mediates the relationship between social attitudes and development, with varying effects depending on whether the attitudes are positive, neutral or negative.

NA ( Table 5) reveals the structural connections between market organisation ( *Q7*), property rights ( *Q9*), welfare policies ( *Q10*) and sustainability outcomes ( *Q12*), reinforcing the link between economic institutions and welfare, as proposed by Acemoglu *et al.* ( 2005; 2012). In the institutional quality network, the rule of law ( *Q3*) is identified as a key factor in maintaining democratic stability ( *Q4*). Two essential findings emerge when social attitudes are incorporated into these networks. First, strongly positive attitudes improve the quality of governance ( *Q13*), in line with Inglehart and Welzel’s (2005) reasoning that progressive values contribute to more responsive institutions. Second, moderately negative attitudes are associated with democratic reforms ( *Q4*), extending Hirschman’s (1970) ‘voice’ theory within a data-driven network framework and highlighting how criticism can both disrupt and renew institutional structures. Furthermore, the institutional quality network appears to function as a selective filter, channelling attitudinal influences towards governance stability. This provides a fresh take on Rodrik *et al.*’s (2004) argument on the primacy of institutions. 

The mediation analysis complements the network analysis, showing that institutional quality mediates the effects of social attitudes on development outcomes ( Table 8). The findings suggest that social attitudes do not directly influence development outcomes but exert their effects through institutional quality variables, with neutral attitudes playing a significant role. Notably, neutral-positive ( *Neu_p*), neutral-mixed ( *Neu_m*) and neutral-negative ( *Neu_n*) attitudes yield significant total effects of 35.14, 25.65 and 17.45, respectively. These total effects are largely driven by substantial indirect effects through institutional quality ( *PC1_iq* and *PC2_iq*). In contrast, strongly positive ( *Pos_s*) and moderately positive ( *Pos_m*) attitudes exhibit smaller yet significant total effects of 12.55 and 4.04, respectively. For *Pos_s* the mediation effect is indirect (10.70), with *Pos_m* showing both direct (2.19) and indirect (1.85) effects. Therefore, while both positive and neutral attitudes have a positive influence on institutional quality, the results underscore the particularly important role of neutral attitudes. This finding can be interpreted in the context of the broader literature on affective polarisation, which suggests that polarisation ultimately undermines institutional quality ( Martínez-España *et al.*, 2024; Romero-Martín *et al.*, 2024). In this context, more neutral positions may play a key role in consensus building and, thus, contribute to institutional resilience. 

Negative attitudes, however, pose something of a challenge. Strongly negative ( *Neg_s*) and moderately negative ( *Neg_m*) attitudes yield significant negative total effects of -10.33 and -11.42, respectively, with negative indirect effects (-8.91; -9.08). This indicates that negative attitudes weaken institutional quality. These results are consistent with Alesina and La Ferrara (2002), who highlight how divisive sentiments erode social cohesion. Nevertheless, we cannot overlook the earlier finding from the NA, which reveals a link between *Neg_m* and democratic stability ( *Q4*). This suggests that, unlike strongly negative attitudes, moderate negativity may drive institutional adaptation when approached constructively. Finally, the strongly neutral group ( *Neu_s*) presents an anomaly. Despite having a significant indirect effect via institutional stability, its total effect is not significant. This suggests that complete disengagement may weaken institutional responsiveness without directly influencing development outcomes.

Our findings offer valuable insights for policymakers, practitioners and international stakeholders. First, our mixed-methods approach highlights key areas for intervention: market resilience, legal robustness and engagement with neutral groups. Second, our results provide a comprehensive roadmap for harnessing social attitudes for sustainable development, with a focus on institutional quality as a key lever. The ultimate goal is to design strategies that collectively empower stakeholders to build governance systems that not only withstand polarisation but thrive on it, harnessing diverse attitudes as a force for sustainable progress. To this end, our results suggest the need to engage neutral groups as strategic allies in this process. Such groups emerge as key actors due to their adaptability, making them key actors in consensus building. This aligns with Black’s (1948) median voter theorem, which emphasises the influence of moderate positions in decision making. Policymakers should target these groups with tailored communication strategies, framing policies in terms of their collective benefits ( Rowbotham *et al.*, 2019) via initiatives such as town hall meetings, media campaigns and community workshops. For example, presenting environmental regulations as opportunities for job creation could motivate neutral groups to support them actively. Similarly, NA ( Table 4) highlights the centrality of market structures ( *Q7*) and legal frameworks ( *Q3*), suggesting the need for structural investments, such as streamlining market regulations and strengthening judicial independence, to maximise the impacts of positive social attitudes. In this context, international organisations like the World Bank could prioritise capacity-building grants to enhance governance stability, ensuring institutions effectively translate social attitudes into tangible policy outcomes. This could involve training programmes for civil servants to improve policy implementation in socially diverse settings, in line with Fukuyama’s (2013) vision of adaptive governance.

However, while our findings offer general guidelines, implementation must be tailored to local contexts, as discussed in Section 3.1 regarding regional variations in social attitudes. To ensure long-term effectiveness, policymakers should develop real-time monitoring systems, such as attitude surveys or governance dashboards, to track societal shifts and adjust interventions accordingly. Working in partnership with NGOs or academic institutions to assess key outcomes, such as policy adoption rates or trust indices, can help to refine strategies and ensure that institutions remain responsive to evolving social attitudes.

## 6. Conclusions and further research

This study advances the ongoing discourse on the determinants of institutional quality and development by highlighting the importance of incorporating social attitudes into economic analysis for a more holistic perspective. The use of innovative methodologies to categorise and analyse attitudes has allowed us to gain a better understanding of how social attitudes shape governance structures and economic performance.

To measure social attitudes, we used data from the World Values Survey, applying set theory and ordinal preferences to rank attitudes on a continuum. The most positive attitudes were defined by support for gender equality and environmental protection and inclusive views on immigration, while the most negative attitudes reflected opposition to these same principles. Institutional quality and development outcomes were assessed using data from Bertelsmann Stiftung’s Transformation Index (BTI). Institutional quality variables serve as proxies for political institutions, following the framework of Acemoglu *et al.* ( 2005; 2012), while development outcomes indicators represent economic institutions and economic performance. Our findings suggest that social attitudes influence development outcomes through institutional quality variables. Positive and, in particular, neutral attitudes are significantly positively associated with institutional quality, whereas negative attitudes are negatively related to institutional quality.

From a methodological standpoint, this study makes a valuable contribution by integrating network analysis with regression-based mediation analysis. This combined approach moves beyond single-method designs by linking data-driven mapping of conditional associations with formal testing of mediated relationships. By capturing both the structure of interconnections among variables and the pathways through which effects are transmitted, the framework offers a multidimensional perspective on how social attitudes influence development outcomes through institutional quality. In practical terms, the findings provide policymakers with a coherent roadmap for leveraging social attitudes to support sustainable development, highlighting institutional quality as a central coordinating mechanism.

The findings of this study lay the groundwork for future research in this area. First, several limitations need to be addressed. The first concerns causality. Our study employs regression-based mediation analysis to distinguish between direct and indirect effects, supplemented by statistical controls, including bootstrapping. This robust methodological approach is in line with best practices for analysing reciprocal relationships ( Efendic *et al.*, 2011). However, the use of instrumental variables could further refine causal estimation. Similarly, while our cross-sectional design is robust, it precludes causal inference over time, a limitation shared with previous studies (e.g. Efendic *et al.*, 2011). Additionally, while our path analysis approach with composite scores is well-suited for small-sample cross-country research and provides robust inference through bootstrapping ( Kelcey, 2019; Yuan *et al.*, 2024), future research with larger samples could employ full latent variable structural equation modelling to further validate these findings. A second limitation is that our analysis focuses primarily on how social attitudes shape institutions and then development outcomes, but institutions also influence social attitudes. The use of longitudinal data would allow for the analysis of these feedback loops between institutions and social attitudes.

Furthermore, regional heterogeneity in social attitudes may limit the generalisability of our findings, particularly for smaller or underrepresented countries. Expanding the analysis to include underrepresented countries would therefore enhance the robustness of the results. Moreover, regional heterogeneity also highlights the need for future research to consider how contextual factors — such as historical legacies, legal traditions, and cultural frameworks — shape the relationships explored in this study. Similarly, further research is needed to identify the factors that strengthen or weaken the role of social attitudes within institutional frameworks. Examining the role of social networks, digital platforms, educational programmes, and civic engagement initiatives could offer valuable insights for responding to the challenges of modern governance. 

Addressing these issues will enhance our understanding of the relationship between social attitudes, institutional quality and development, ultimately leading to more effective governance and sustainable economic progress.

## Data Availability

1. Network analysis was conducted using the JASP© package, while regression-based mediation analysis was performed in STATA 18. All relevant files, including executable and result files, are available via Zenodo for review and reproducibility:
https://doi.org/10.5281/zenodo.15092462 ( Liashenko & Caraballo Pou, 2025a). 2. via the following supplementary repository:
https://doi.org/10.5281/zenodo.15373260 ( Liashenko & Caraballo Pou, 2025c). 3. The original datasets used in this study are publicly available through the World Values Survey and Bertelsmann Transformation Index project websites. We do not have the right to redistribute this data directly. Researchers may access the datasets at:
- World Values Survey Wave 7 (2017–2022), Version 4.0.0: https://doi.org/10.14281/18241.18
- Bertelsmann Transformation Index (BTI) 2024 Scores: https://bti-project.org Due to redistribution restrictions associated with the BTI, we are unable to provide the original .jasp project file containing BTI variables. Sharing these data, even indirectly, such as embedded within an analysis file, would violate BTI’s data usage policy. Moreover, .jasp files are not standalone executable scripts: all data and model specifications are embedded within the file. Without the original dataset, the file cannot be interpreted or executed meaningfully. To ensure transparency and reproducibility, we provide:
- a replication guide detailing the full procedure for Bayesian network estimation in JASP; - a CSV template with variable headers, allowing researchers to insert BTI data obtained directly from https://bti-project.org and replicate the analysis accordingly. The extended data comprises a structured appendix А that contains all supplementary analyses and results referenced throughout the article, supporting complete models transparency, replication, and diagnostic evaluation: The complete appendix is available in the data repository via Zenodo: https://doi.org/10.5281/zenodo.18374589 ( Liashenko & Caraballo Pou, 2026).
i. Appendix A.I – Social Attitude Typology and Network Models. Begins with a country-level table of aggregated social attitude types, derived from 27 ordinal combinations of individual responses, grouped into eight composite categories (e.g., Neg_s, Neu_p, Pos_m). This section also presents results from four network models, estimated via the EBICglasso algorithm within JASP’s Bayesian Network module. While the models are technically undirected partial correlation networks, they are grounded in Bayesian estimation principles, incorporating: edge inclusion probabilities, expected influence metrics, and model parsimony penalised via the Extended Bayesian Information Criterion (EBIC). These features place the networks within a Bayesian modelling framework, despite regularisation. The four networks include: - Development Outcomes Network - Combined Institutional Quality and Development Outcomes Network - Development Outcomes with Attitudes (DOA) Network - Institutional Quality and Attitudes (IQA) Network Each model includes sparsity measures, centrality scores (strength, closeness, betweenness, expected influence), and graphical figures illustrating key variables and hubs.
ii. Appendix A.II – Principal Component Analysis (PCA) contains results from two PCA models based on institutional and development indicators, which inform the selection of latent constructs for regression-based mediation analysis. iii. Appendix A.III – Multicollinearity Diagnostics Reports VIF analysis results for BTI indicators, identifying multicollinearity risks that may affect mediation analysis stability. In addition to the main appendix, a separate Zenodo repository provides replication materials for the network models developed in JASP. This includes variable templates, a step-by-step analysis guide, and supporting files for reconstructing network models using BTI indicators and aggregated attitude typologies. While the .jasp project file could not be shared due to data licensing restrictions, all necessary components for reproducing the analysis are provided. https://doi.org/10.5281/zenodo.15373406 ( Liashenko & Caraballo Pou, 2025e) Data are available under Creative Commons Attribution 4.0 International This study used secondary data from Wave 7 of the World Values Survey and the Bertelsmann Stiftung’s Transformation Index. We reviewed relevant reporting standards from the EQUATOR Network. Given the nature of the data and study design, the STROBE and SAGER guidelines were partially applicable. Relevant reporting guidelines from the EQUATOR Network were reviewed. Due to the data's secondary nature and this study's analytical focus, the STROBE and SAGER guidelines were not fully applicable. The variable related to gender equality (Q33) was adopted from the standardised World Values Survey questionnaire and was employed exclusively as a proxy indicator of societal attitudes towards contested public issues; gender was not a research focus. Completed STROBE and SAGER checklists have been deposited in the ZENODO repository and are referenced below: https://doi.org/10.5281/zenodo.15265622 ( Liashenko & Caraballo Pou, 2025b).

## Footnotes

[1] The definition of each institutional quality and development outcomes variable, along with their component parts, methodology and aggregation methods, is fully detailed at https://bti-project.org/en/methodology.

## Software availability statement

The regression-based mediation analysis in this article was performed using STATA version 18, a proprietary statistical software ( https://www.stata.com). For those without access to STATA, equivalent analyses can be performed using open-source alternatives such as R ( https://cran.r-project.org).

Network Analysis (NA) was conducted using JASP version 0.18.1, an open-source graphical statistical software based on R and Python, freely available at https://jasp-stats.org/. (see **Note on data restrictions** section for details)
