# Supplementary material for: From Social Attitudes to Outcomes: The Role of Institutional Quality in Development
Source: Open Res Eur. 2026 Feb 14;5:146. Originally published 2025 May 28. [Version 2] doi: 10.12688/openreseurope.20302.2 (PMC12853015; doi:10.12688/openreseurope.20302.2)
Supplement: Peer review history

# Reviewer report: Reviewer response for version 2

**DOI:** 10.21956/openreseurope.24876.r69605

**Published:** 2026-03-18

**Authors:** Joshua Adeyemi Afolabi

**Affiliations:** 1 Technische Universität Ilmenau, Thüringen, Germany

All comments have been addressed.

If applicable, is the statistical analysis and its interpretation appropriate?

Partly

Is the study design appropriate and is the work technically sound?

Yes

Is the work clearly and accurately presented and does it engage with the current literature?

Yes

Are the conclusions drawn adequately supported by the results?

Partly

Are sufficient details of methods and analysis provided to allow replication by others?

Yes

Are all the source data and materials underlying the results available?

Yes

Reviewer Expertise:

My research area is Economics.

I confirm that I have read this submission and believe that I have an appropriate level of expertise to confirm that it is of an acceptable scientific standard.

# Reviewer report: Reviewer response for version 2

**DOI:** 10.21956/openreseurope.24876.r69607

**Published:** 2026-02-19

**Authors:** Javier Barbero

**Affiliations:** 1 Department of Economics, Universidad Autonoma de Madrid Facultad de Ciencias Economicas y Empresariales (Ringgold ID: 88092), Madrid, Community of Madrid, Spain

No further comments.

If applicable, is the statistical analysis and its interpretation appropriate?

Yes

Is the study design appropriate and is the work technically sound?

Yes

Is the work clearly and accurately presented and does it engage with the current literature?

Yes

Are the conclusions drawn adequately supported by the results?

Yes

Are sufficient details of methods and analysis provided to allow replication by others?

Partly

Are all the source data and materials underlying the results available?

Partly

Reviewer Expertise:

My research area is Economics. I can only assess whether the manuscript is written in scientific form and follows the scientific method. I do not know the methodology used, and I cannot assess whether its use was appropriate. Another reviewer, an expert in Bayesian Network Analysis (BNA) and Structural Equation Modeling (SEM), should assess section 4.

I confirm that I have read this submission and believe that I have an appropriate level of expertise to confirm that it is of an acceptable scientific standard.

# Reviewer report: Reviewer response for version 1

**DOI:** 10.21956/openreseurope.21967.r67823

**Published:** 2026-01-28

**Authors:** Joshua Adeyemi Afolabi

**Affiliations:** 1 Technische Universität Ilmenau, Thüringen, Germany

The paper “From Social Attitudes to Outcomes: The Role of Institutional Quality in Development” is well-written. Using data from Wave 7 of the World Values Survey, it reveals that institutional quality is a key bridge between social attitudes and development outcomes, and it mediates these effects in most cases. Neutral-positive and mixed-neutral attitudes yield the most potent positive indirect effects, while negative attitudes are associated with institutional weakening and lower development performance.

 However, some of the variables used for the empirical analysis are highly correlated (see Appendix A.III.1). In particular, Pos_m and Neg_m and Neu_p and Neu_m exhibit very strong correlations, which exceed the acceptable threshold of 0.8. These correlation coefficients imply that SEM estimates may suffer from weak identification and unstable path coefficients. Thus, authors should reduce redundancy by consolidating or re-specifying highly correlated sentiment variables, for example, through latent factors or variable selection. Alternatively, robustness checks should be done to verify whether the results will remain the same. Besides, the correlation matrix should include not only social attitude indicators but all key variables: social attitude, institutional quality, and development outcomes.

 In addition, the reported VIF values are extraordinarily large in Appendix A.III.3, with a maximum above 70 and a mean above 30. This level of multicollinearity far exceeds conventional thresholds and implies that coefficient estimates in the SEM model are likely unstable, imprecise, and sensitive to small specification changes. Authors should consider reducing the number of highly correlated regressors through variable aggregation, dimension reduction, or re-specifying the model to use latent constructs rather than individual sentiment measures. This will obviously change the current narratives in the paper.

 On Section 3.2 (page 9), the paper reads, “These two elements can be analysed using the Development Outcomes indicators, which include data on the quality of economic institutions, such as Private Property and the Organisation of the Market and Competition.” This implies that the indicators of institutional quality and development outcomes are empirically overlapping, which weakens their distinction in the estimation process. This should be resolved to ensure the credibility of the results, as the lack of a clear empirical separation will propagate through all subsequent estimation exercises and undermine the interpretation of the findings.

If applicable, is the statistical analysis and its interpretation appropriate?

Partly

Is the study design appropriate and is the work technically sound?

Yes

Is the work clearly and accurately presented and does it engage with the current literature?

Yes

Are the conclusions drawn adequately supported by the results?

Partly

Are sufficient details of methods and analysis provided to allow replication by others?

Yes

Are all the source data and materials underlying the results available?

Yes

Reviewer Expertise:

My research area is Economics.

I confirm that I have read this submission and believe that I have an appropriate level of expertise to confirm that it is of an acceptable scientific standard, however I have significant reservations, as outlined above.

# Response

**Published:** 2026-02-22

**Authors:** Mª Ángeles Caraballo

**Affiliations:** Economía e Historia Económica, University of Seville, Seville, Andalusia, Spain

We are most grateful to *Joshua Adeyemi Afolabi* for the careful and constructive reading of our manuscript. The reviewer’s comments are substantive and reflect genuine expertise in econometrics and applied institutional analysis. We note that this review was prepared in response to Version 1, whereas Version 2 of the manuscript was submitted independently and has since been published. Nevertheless, we welcome the opportunity to respond to each point in full, and trust that the revisions already incorporated into Version 2 will satisfy the reviewer’s principal concerns. We address each comment in turn below. **Reviewer’s Comment 1: High Inter-Correlations Among Attitude Variables** *The reviewer observes that several attitude variables exhibit very strong pairwise correlations in Appendix A.III.1 — specifically between Pos_m and Neg_m, and between Neu_p and Neu_m — with values exceeding the conventional threshold of 0.8. The reviewer suggests that these correlations may induce weak identification and unstable path coefficients in the SEM framework, and recommends either consolidating or re-specifying the correlated variables through latent factors or variable selection, or conducting robustness checks. The reviewer also requests that the correlation matrix be expanded to include not only attitude indicators but all key variables: social attitudes, institutional quality, and development outcomes.* **Authors’ Response:** We thank the reviewer for raising this important methodological point. We wish to clarify that the high intercorrelations identified in Appendix A.III.1 are both expected and explicitly addressed in the analytical design of Version 2. The attitude variables are constructed as country-level proportions of respondents belonging to each of eight attitudinal categories. Because these proportions are derived from a common population and sum to unity within each country, a degree of negative or positive correlation among them is a mathematical and empirical inevitability rather than a modelling deficiency. The strong negative correlation between, for instance, Pos_m and Neg_m simply reflects the compositional constraint inherent in proportion data: as one attitudinal share increases, others must correspondingly decrease. The solution adopted in Version 2, which supersedes the SEM specification that informed the reviewer’s concerns, is precisely designed to circumvent the multicollinearity problem. Rather than estimating a single structural equation in which all eight attitude variables enter simultaneously as regressors, we conduct separate mediation analyses for each attitude variable. This means that each model contains only one attitudinal predictor (X _a_), alongside the two PCA-based composite mediators (PC1_iq and PC2_iq) and the outcome composite (PC1_out). The multicollinearity issue that would arise from entering all attitude variables jointly is thus precluded by construction. This modelling strategy is explicitly stated in Section 4.2 of Version 2, with reference to standard practice in regression-based mediation analysis (Hayes, 2017). Regarding the composition of the correlation matrix: the full multicollinearity diagnostics for the BTI indicators, which informed the decision to construct PCA-based composites rather than entering all institutional and development variables individually, are reported in Appendix A.III. The PCA procedure itself, as applied to the institutional quality and development outcomes variables, produces orthogonal components by construction, thereby ensuring that the composite mediators (PC1_iq, PC2_iq) and the outcome variable (PC1_out) are empirically uncorrelated with one another. A comprehensive correlation table spanning all raw variables simultaneously would, in our view, be less informative in the context of this modelling strategy than the combination of the diagnostic VIF analysis and the PCA output already provided; however, we would be happy to include such a table in the Appendix should the reviewer consider it essential for transparency. **Reviewer’s Comment 2: Extraordinarily Large VIF Values** *The reviewer notes that the VIF values reported in Appendix A.III.3 are very large (maximum exceeding 70, mean exceeding 30), well beyond conventional thresholds, and infers that coefficient estimates in the SEM are likely unstable, imprecise, and sensitive to specification changes. The reviewer recommends reducing the number of highly correlated regressors through variable aggregation, dimension reduction, or re-specifying the model to use latent constructs.* **Authors’ Response:** We are grateful for this comment and take the opportunity to clarify the precise diagnostic role served by the VIF analysis in Appendix A.III.3, which appears to have been interpreted as pertaining to the estimation model itself rather than to its motivating diagnostics. The VIF values reported in Appendix A.III.3 were computed by regressing each BTI indicator against all remaining BTI indicators simultaneously, that is, in a saturated auxiliary regression including all institutional quality and development outcomes variables at once. This exercise was conducted not as a model in its own right, but as a diagnostic to demonstrate the severity of multicollinearity among the raw BTI variables, thereby justifying our subsequent decision to employ PCA to reduce these variables to a small set of orthogonal composites before proceeding to mediation analysis. In other words, the high VIF values are not symptoms of a problem in our estimation; rather, they constitute the empirical evidence that motivated our dimensionality-reduction strategy. The mediation models estimated in Section 4.2 of Version 2 do not enter these raw BTI variables as simultaneous regressors. Each model contains three explanatory quantities: one attitudinal variable (entered individually, as noted in our response to Comment 1 above), and the two PCA-derived institutional composites (PC1_iq and PC2_iq), which are orthogonal by construction. The multicollinearity diagnosed in the auxiliary VIF regression is therefore not present in the models from which our substantive inferences are drawn. The reviewer’s recommendation to employ latent constructs or dimension reduction is, in fact, precisely what Version 2 implements through the PCA approach. The retained components account for 89% of the variance in the institutional quality variables and 83% of the variance in the development outcomes variables (Tables A.II.1 and A.II.2), and are supported by balanced loading structures (Table 7) and standard scree plot criteria. This approach follows established methodological guidance on composite construction for multidimensional indicators (Mazziotta & Pareto, 2013, 2018). We trust that this clarification resolves the reviewer’s concern regarding estimation instability. **Reviewer’s Comment 3: Empirical Overlap Between Institutional Quality and Development Outcomes** *The reviewer draws attention to the passage in Section 3.2* *, where the paper states that the Development Outcomes indicators include data on the quality of economic institutions, such as Private Property and the Organisation of the Market and Competition. The reviewer interprets this as indicating that the institutional quality and development outcomes constructs overlap empirically, which would weaken their analytical distinction in the estimation process and undermine the interpretation of the mediated pathway.* **Authors’ Response:** We appreciate this perceptive observation, which touches on a genuine theoretical complexity in the literature on institutional economics. We welcome the opportunity to clarify both the conceptual basis for our variable categorisation and the empirical strategy that ensures adequate separation between the mediator and outcome constructs. The categorisation of BTI variables into ‘institutional quality’ and ‘development outcomes’ is related to the analytical framework developed by Acemoglu, Johnson, and Robinson (2005, 2012). This framework distinguishes between political institutions (characterised by checks on power, rule of law, democratic participation, and governance quality) and economic institutions and outcomes, including market organisation, private property rights, and levels of socioeconomic development. Although Private Property and the Organisation of the Market and Competition are indeed captured under the Development Outcomes heading in the BTI framework, their inclusion there reflects the BTI’s conceptualisation of these as outputs of the institutional environment rather than as constituents of governance itself. This distinction, while imperfect, is consistent with usage in the comparative political economy literature and is explicitly acknowledged in the paper. More importantly, the empirical separation between the mediator and the outcome in our models is achieved not through an assumption of conceptual non-overlap in the raw data, but through PCA. Principal Component Analysis extracts orthogonal dimensions of variation from each variable set independently. PC1_iq and PC2_iq are derived exclusively from the institutional quality variables and capture the primary axes of governance-related variance, whilst PC1_out is derived independently from the development outcomes variables and captures the dominant dimension of economic performance variation. Because the two PCA models are estimated separately, the resulting composites are empirically distinct even where the underlying raw indicators share some conceptual ground. The mediation model, therefore, tests whether variation in the governance-related composite (M) transmits the effect of social attitudes (X) onto the economic performance composite (Y). This represents a structurally coherent and identifiable pathway. We acknowledge that the conceptual boundary between political and economic institutions in the BTI is not perfectly sharp. We have noted this as a limitation of the current study in Section 6 of Version 2, and identify it a productive direction for future research. **Concluding Remarks** We hope that the above responses demonstrate that the reviewer’s substantive concerns regarding multicollinearity, VIF diagnostics, and the empirical separation of constructs are directly addressed by the modelling strategy adopted in Version 2 of the manuscript. The transition from a single-equation SEM framework to a series of separate regression-based mediation models with PCA-derived composites was motivated precisely by the empirical challenges the reviewer has identified and reflects best practice for small-sample cross-country research (Kelcey, 2019; Savalei, 2019; Yuan et al., 2024). We remain grateful for the reviewer’s engagement with our work and are happy to provide any further clarification that may be required.

# Reviewer report: Reviewer response for version 1

**DOI:** 10.21956/openreseurope.21967.r67668

**Published:** 2026-01-21

**Authors:** Samitha Udayanga

**Affiliations:** 1 Bremen University, Bremen, Germany

While the research question is relevant and the paper addresses an important topic, there are **significant methodological concerns** with the Structural Equation Modeling (SEM) implementation that undermine the validity of the findings.

 The authors use Principal Component Analysis (PCA) to create "latent variables" (PC1_iq, PC2_iq, PC1_out) which they then use in their SEM. 

- PCA creates orthogonal components based on maximum variance, not theoretically-grounded latent constructs
- Proper SEM requires a measurement model where latent variables are defined by their indicators through confirmatory factor analysis (CFA)
- The loadings in Table 7 are PCA loadings, not factor loadings from a proper measurement model
- This approach bypasses the fundamental distinction between observed and latent variables that is central to SEM

**Recommendation**: Conduct proper CFA to establish measurement models for Institutional Quality and Development Outcomes before testing structural relationships.

**The Presented "SEM" is Actually Multiple Regression**

 The three equations (1-3) presented are **regression equations**, not a genuine SEM specification:

- No measurement model is specified
- No error term correlations or covariances are modeled
- The approach of running separate models for each attitude variable (to avoid multicollinearity) means this is **eight separate regression-based mediation analyses**, not SEM
- True SEM would test all relationships simultaneously in one integrated model

**Recommendation**: Either reframe this as "regression-based mediation analysis" or implement proper SEM with full model specification.

 The paper lacks critical elements of SEM reporting:

- **No model fit indices** (CFI, TLI, RMSEA, SRMR, chi-square)
- No discussion of measurement model evaluation
- No assessment of discriminant/convergent validity
- No path diagram showing the full structural model

The authors justify running separate models due to multicollinearity among attitude variables. However:

- Proper SEM can handle correlated predictors
- Running separate models prevents comparison of relative effects
- This approach fragments what should be a unified theoretical model
- Standard diagnostic procedures (VIF, tolerance) could address collinearity concerns within a single model

The "Bayesian Network Analysis" using EBICglasso is actually regularized precision matrix estimation producing "partial correlation structures, not classic causal Bayesian graphs" (authors' own words). The terminology is misleading.

 The 27-category scheme collapsed to 8 categories (Tables 2-3) seems unnecessarily complex. The theoretical justification linking Arrow's impossibility theorem to this classification is tenuous.

 Also, further review is needed for BNA. I am not commenting on BNA, as my expertise on BNA is limited. 

 comments given pdf to authors.

*https://openreseurope-files.f1000.com/linked/266359.ID_20302_comments_given_pdf_to_authors._20302_-_ma_angeles_caraballo.pdf*

If applicable, is the statistical analysis and its interpretation appropriate?

Partly

Is the study design appropriate and is the work technically sound?

Partly

Is the work clearly and accurately presented and does it engage with the current literature?

Partly

Are the conclusions drawn adequately supported by the results?

Partly

Are sufficient details of methods and analysis provided to allow replication by others?

No

Are all the source data and materials underlying the results available?

Partly

Reviewer Expertise:

I am a sociologist and was able to assess most sections of this paper; however, another expert should review the BNA component.

I confirm that I have read this submission and believe that I have an appropriate level of expertise to confirm that it is of an acceptable scientific standard, however I have significant reservations, as outlined above.

# Response

**Published:** 2026-01-26

**Authors:** Mª Ángeles Caraballo

**Affiliations:** Economía e Historia Económica, University of Seville, Seville, Andalusia, Spain

**Response to Reviewer Comments** We would like to express our sincere gratitude to the Reviewer for the thorough and constructive evaluation of our manuscript. The comments have been invaluable in improving the clarity, precision, and transparency of our work. Below, we provide a detailed point-by-point response to each concern raised. All substantive revisions have been incorporated into the revised manuscript, with changes highlighted in red. ***Comment 1: Use of PCA Instead of CFA for Latent Variables*** **Reviewer’s concern:** “PCA creates orthogonal components based on maximum variance, not theoretically-grounded latent constructs. Proper SEM requires a measurement model where latent variables are defined by their indicators through confirmatory factor analysis (CFA).” **Response:** We appreciate this methodological observation and have carefully considered the distinction between PCA-derived composites and CFA-based latent variables. We acknowledge that PCA and CFA serve different purposes: CFA tests a priori measurement structures, whereas PCA is a data-reduction technique that maximises explained variance. However, our use of PCA is intentional and methodologically justified given our research objectives and sample constraints. **First**, our primary aim is not to confirm a pre-specified measurement model but rather to construct parsimonious composite indicators that capture the dominant dimensions of institutional quality and development outcomes. This approach aligns with established methodological guidance on composite index construction. Mazziotta and Pareto (2016) provide a comprehensive review of methods for constructing composite indices, demonstrating that PCA is particularly well-suited when the objective is to synthesise multidimensional information into summary measures that preserve maximum variance from the original indicators. The BTI indicators are already theoretically grounded aggregates developed by the Bertelsmann Stiftung using expert assessments and standardised methodologies. PCA further reduces dimensionality while retaining as much information as possible from these pre-validated indicators. **Second**, our PCA application follows best practices for measuring multidimensional constructs, such as institutional quality and development outcomes. Mazziotta and Pareto (2018), in their influential methodological paper on the use of PCA for measuring well-being, establish clear criteria for when PCA-based composite indicators are appropriate: (i) when individual indicators are conceptually related and expected to measure facets of a common underlying construct; (ii) when the first principal component(s) explain a substantial proportion of total variance, indicating strong convergence among indicators; and (iii) when loadings are relatively balanced, suggesting that no single indicator dominates the composite. Our data meet all three criteria. For Institutional Quality, PC1 and PC2 account for 89% of the total variance, with balanced loadings across governance, democratic quality, and rule-of-law indicators. For Development Outcomes, PC1 accounts for 83% of variance with uniformly positive loadings across all economic and social indicators. These results provide strong empirical support for the unidimensionality of our constructs and the validity of our composite measures. **Third**, our sample size (n=59 countries) imposes significant constraints on full latent-variable SEM with measurement models. Recent methodological literature demonstrates that path analysis with composite scores offers superior performance in small-sample settings:

- Yuan et al. (2024) demonstrate that path analysis with weighted composites yields greater signal-to-noise ratios than full SEM for mediation analysis, particularly in smaller samples.
- Kelcey (2019) shows that composite-based approaches exhibit superior small-sample properties.
- Savalei (2019) confirms that such methods produce estimates comparable to those from full SEM while being more robust to model misspecification.

**Fourth**, the high proportion of variance explained by the retained components (89% for Institutional Quality; 83% for Development Outcomes) exceeds conventional thresholds recommended in the composite indicators literature. As Mazziotta and Pareto (2016) note, when the first component(s) capture such a substantial share of total variance, PCA-derived composites provide reliable summary measures of the underlying multidimensional construct. The loading structures in Table 7 further confirm that our composites are well-balanced representations of the constituent domains rather than being driven by any single indicator. We have revised the manuscript to clarify our methodological approach, explicitly framing it as “path modelling with composite scores” rather than latent variable SEM, and have added the relevant methodological references (Yuan et al., 2024; Kelcey, 2019; Savalei, 2019; Mazziotta & Pareto, 2016, 2018) to support this choice. **Revisions made:** Section 4.2 has been substantially revised to clarify the analytical framework and provide methodological justification for the composite-based approach. References to Mazziotta and Pareto (2016, 2018) have been added to support the use of PCA for composite index construction. ***Comment 2: The Presented “SEM” is Actually Multiple Regression*** **Reviewer’s concern:** “The three equations (1-3) presented are regression equations, not a genuine SEM specification. No measurement model is specified. No error term correlations or covariances are modelled. The approach of running separate models for each attitude variable (to avoid multicollinearity) means this is eight separate regression-based mediation analyses, not SEM.” **Response:** We fully accept this characterisation and have revised the manuscript accordingly. The Reviewer is correct that our analysis constitutes regression-based mediation analysis rather than full structural equation modelling with latent variables. We have made the following terminological and framing changes throughout the manuscript:

1. **Abstract:** Changed “Structural Equation Modelling (SEM)” to “mediation analysis through path modelling with composite scores”
2. **Section 4.2 title:** Changed from “Structural Equation Model” to “Mediation Analysis Using Path Modelling”
3. **Throughout the text:** Replaced references to “SEM” with “regression-based mediation analysis” or “path modelling with composite scores”
4. **Discussion and Conclusions:** Updated methodological descriptions accordingly

We have also explicitly acknowledged that our approach involves “path analysis with composite scores rather than full latent variable structural equation modelling” and provided methodological justification for this choice, given our sample size constraints. Regarding the decision to run separate models for each attitude variable: this approach was adopted deliberately to address multicollinearity among the attitude variables (documented in Table A.III.1 in Appendix A.III), following standard practice in mediation analysis when predictors are highly correlated (Hayes, 2017). While we acknowledge that this prevents direct comparison of relative effect sizes across attitudes within a single model, it ensures that the estimated coefficients for each attitude are not distorted by collinearity. We have clarified this rationale in the revised manuscript. **Revisions made:** Comprehensive terminological revisions throughout the manuscript; added methodological justification in Section 4.2. ***Comment 3: Missing Model Fit Indices*** **Reviewer’s concern:** “The paper lacks critical elements of SEM reporting: No model fit indices (CFI, TLI, RMSEA, SRMR, chi-square), No discussion of measurement model evaluation, No assessment of discriminant/convergent validity, No path diagram showing the full structural model.” **Response:** We appreciate this observation and have addressed it by clarifying why traditional SEM fit indices are not applicable to our analytical approach. Our mediation models are *just-identified* (degrees of freedom = 0), meaning that the model-implied covariance matrix perfectly reproduces the observed covariance matrix by construction. In such cases, fit indices such as CFI, TLI, RMSEA, and SRMR are either undefined or trivially perfect, and chi-square equals zero. This is a well-known characteristic of just-identified path models and is explicitly noted in standard references (e.g., Kline, 2016). For regression-based mediation analysis with composite scores, the appropriate evaluation criteria are:

1. *Bootstrapped confidence intervals* for indirect effects (which we report using 5,000 replications)
2. *Explained variance* for each equation
3. *Statistical significance* of path coefficients

These criteria are standard for regression-based mediation analysis as outlined by Hayes (2017) and have been applied throughout our analysis. We have added the following clarification to Section 4.2: “As this analysis employs path modelling with composite scores rather than latent variable SEM with measurement models, traditional fit indices (CFI, TLI, RMSEA, SRMR) are not applicable to our model specification. Our just-identified mediation models (degrees of freedom = 0) are assessed through bootstrapped confidence intervals for indirect effects and explained variance for each equation, which are standard criteria for regression-based mediation analysis (Hayes, 2017).” **Revisions made:** Added explicit explanation in Section 4.2 regarding the inapplicability of traditional fit indices and the appropriate evaluation criteria for our analytical approach. ***Comment 4: Handling of Multicollinearity*** **Reviewer’s concern:** “The authors justify running separate models due to multicollinearity among attitude variables. However, Proper SEM can handle correlated predictors; Running separate models prevents comparison of relative effects; This approach fragments what should be a unified theoretical model.” **Response:** We acknowledge the trade-offs inherent in our approach and have provided additional justification in the revised manuscript. While it is true that SEM can accommodate correlated predictors, the degree of multicollinearity among our attitude variables (documented in Table A.III.1, Appendix A.III) is sufficiently high to warrant concern about coefficient stability and interpretability. In such circumstances, running separate models is a recognised and widely-used strategy in mediation analysis (Hayes, 2017). Moreover, our theoretical framework does not posit that different attitude types compete with one another in influencing institutional quality; rather, each attitude category represents a distinct segment of the population with its own pathway to institutional and developmental outcomes. The research question is not “which attitude has the strongest effect?” but rather “how does each type of attitude relate to development outcomes through institutional quality?” For this purpose, separate mediation analyses are appropriate and provide clearer, more interpretable results. We have added explicit reference to the multicollinearity diagnostics in the main text and clarified the rationale for our analytical strategy: “Given that our explanatory variables represent distinct but interrelated aspects of a broader construct (Tables A.III.1, A.III.2, and A.III.3 in Appendix A.III), we perform separate mediation analyses for each attitude to mitigate multicollinearity. This approach ensures that the effect of each explanatory variable on PC1_out is accurately estimated, without distortion from high correlations among the explanatory variables.” **Revisions made:** Enhanced justification for the separate-models approach in Section 4.2, with explicit reference to multicollinearity diagnostics in the Appendix. ***Comment 5: Misleading “Bayesian Network Analysis” Terminology*** **Reviewer’s concern:** “The ‘Bayesian Network Analysis’ using EBICglasso is actually regularised precision matrix estimation producing ‘partial correlation structures, not classic causal Bayesian graphs’ (authors’ own words). The terminology is misleading.” **Response:** We accept this critique and have substantially revised the terminology throughout the manuscript to avoid any potential confusion. The term “Bayesian” in our original manuscript referred specifically to the *Extended Bayesian Information Criterion (EBIC)* used for model selection within the EBICglasso algorithm, not to Bayesian probabilistic network modelling. We acknowledge that this could be misinterpreted and have made the following changes:

1. **Abstract:** Changed “Bayesian Network Analysis (BNA)” to “partial correlation network modelling using the EBICglasso algorithm with the Extended Bayesian Information Criterion (EBIC) for model selection”
2. **Section 4.1 title:** Changed from “Bayesian Network Analysis” to “Network Analysis”
3. **Throughout the text:** Replaced “BNA” with “NA” (Network Analysis) or “network modelling”
4. **Added clarification:** “In this context, the term ‘Bayesian’ refers exclusively to the information criterion used for model selection within the EBICglasso algorithm, rather than to Bayesian probabilistic network modelling. This terminology follows the JASP Network module documentation.”
5. **Added explicit statement:** “It is important to note that the estimated networks do not provide probabilistic measures of association. Instead, edges represent regularised partial correlation coefficients, indicating the presence and relative strength of conditional dependencies among variables.”

**Revisions made:** Comprehensive terminological revisions in Abstract, Section 4.1, and throughout the manuscript; added clarifying statements about the nature of the network estimation approach. ***Comment 6: Complexity of the 27-Category Classification Scheme*** **Reviewer’s concern:** “The 27-category scheme collapsed to 8 categories (Tables 2-3) seems unnecessarily complex. The theoretical justification linking Arrow’s impossibility theorem to this classification is tenuous.” **Response:** We appreciate this feedback and have revised the relevant section to clarify our methodological rationale. **Regarding the classification scheme:** The 27 attitude combinations arise naturally from the cross-tabulation of three survey questions, each with three response categories (positive, neutral, negative). This represents the complete combinatorial space of possible response patterns. Aggregating these into 8 categories reduces complexity while preserving meaningful distinctions along the positive-neutral-negative continuum. The grouping logic is transparent and reproducible: strongly positive attitudes require positive responses across all three dimensions, strongly negative attitudes require negative responses across all three, and intermediate categories capture various combinations. This approach is consistent with standard practices in survey research and attitude measurement, where multidimensional response patterns are often aggregated into interpretable typologies. The 8-category scheme balances parsimony with the ability to distinguish theoretically meaningful attitude profiles. **Regarding the reference to Arrow’s Impossibility Theorem:** We accept that our original framing may have overstated the connection to social choice theory. We have revised the text to clarify that we draw on the *methodological tradition* of working with ordinal preferences, rather than directly applying social choice theorems: “ *The grouping of attitudes draws on concepts from set theory and ordinal preferences. While we do not directly apply social choice theorems, our approach follows the methodological tradition in economics and sociology of working with ordinal rankings without imposing arbitrary numerical scales.”* The references to Arrow (1951), Black (1948), and Bergstrom & Goodman (1973) are retained to acknowledge the intellectual heritage of ordinal preference analysis, but we no longer claim a direct theoretical derivation from these frameworks. **Revisions made:** Section 3.1 has been revised to soften the link to social choice theory while maintaining the methodological justification for ordinal grouping. ***Comment 7: Insufficient Details for Replication*** **Reviewer’s concern:**  “Are sufficient details of methods and analysis provided to allow replication by others? No”  “Are all the source data and materials underlying the results available? Partly” **Response:** We respectfully disagree with this assessment and would like to draw the Reviewer’s attention to the extensive replication materials we have provided. **Data Availability:** The original datasets used in this study are publicly available: - World Values Survey Wave 7 (2017–2022): https://doi.org/10.14281/18241.18 - Bertelsmann Transformation Index (BTI) 2024: https://bti-project.org We cannot redistribute BTI data directly due to licensing restrictions, but we have provided: - A CSV template with variable headers allowing researchers to insert BTI data obtained directly from the source - Step-by-step replication guides for all analyses **Analysis Files and Outputs:** We have deposited comprehensive replication materials in multiple Zenodo repositories:  

1. **Main supplementary files** (Zenodo: https://doi.org/10.5281/zenodo.15092462):

- All STATA executable files for mediation analysis
- Result files

 

1. **STATA output logs** (Zenodo: https://doi.org/10.5281/zenodo.15373260):

- Complete estimation logs in PDF format for researchers without STATA access

 

1. **Network analysis replication materials**

(Zenodo: https://doi.org/10.5281/zenodo.15373406):

- Variable templates
- Step-by-step analysis guide for JASP
- Supporting files for reconstructing network models

 

1. **Extended Appendix A** (Zenodo: https://doi.org/10.5281/zenodo.15373762):

- Complete PCA results with eigenvalues, variance explained, and scree plots
- All network models with sparsity measures and centrality scores
- Multicollinearity diagnostics (VIF analysis)
- Country-level attitude typology data

 
**Methodological Documentation:** The manuscript and appendix provide: - Complete specification of all equations (Equations 1-3) - Detailed description of variable construction (Tables 2-3) - PCA loadings for all components (Table 7) - Full mediation results with all coefficients (Tables 8-9) - Bootstrap specifications (5,000 replications) We believe these materials meet—and in many respects exceed—standard requirements for reproducibility in cross-country empirical research. If the Reviewer has specific concerns about particular aspects of the analysis that require additional documentation, we would be pleased to address them. **Revisions made:** The Data Availability and Extended Data sections have been reviewed and enhanced for clarity; all Zenodo links have been verified and remain accessible. ***Comment 8: Reviewer’s Self-Acknowledged Limitation Regarding BNA*** **Reviewer’s statement:** “Also, further review is needed for BNA. I am not commenting on BNA, as my expertise on BNA is limited.” **Response:** We appreciate the Reviewer’s transparency regarding their expertise. We note that, while the Reviewer has not provided substantive comments on the network analysis component, we have nonetheless revised the terminology and framing, as detailed in our response to Comment 5, to ensure clarity for all readers. The network analysis serves an exploratory function in our study, identifying conditional dependencies among variables to inform the subsequent mediation analysis. We have clarified this role in the revised manuscript and ensured that the methodological description is accessible to readers from various disciplinary backgrounds. **Summary of Revisions** **Section** **Nature of Revision** Abstract Revised methodological terminology (network modelling, path modelling with composite scores) Section 1 (Introduction) Updated analytical framework description Section 3.1 Softened link to social choice theory; clarified ordinal grouping rationale Section 4.1 Changed “Bayesian Network Analysis” to “Network Analysis”; added clarifications about EBICglasso Section 4.2 Comprehensive revision: reframed as “Mediation Analysis Using Path Modelling”; added methodological justification for composite-based approach; explained inapplicability of fit indices; added new references Section 5 (Discussion) Updated methodological descriptions Section 6 (Conclusions) Updated methodological descriptions; acknowledged limitations and directions for future research References Added: Yuan et al. (2024), Kelcey (2019), Savalei (2019), Kline (2016), Muthén & Muthén (2002), Mazziotta & Pareto (2016, 2018) Data availability Reviewed and clarified; verified all repository links   Concluding Remarks We trust that these revisions adequately address the Reviewer’s concerns while maintaining the integrity and contribution of our research. The revised manuscript now provides:

1. **Clearer methodological framing** that accurately describes our analytical approach as path modelling with composite scores rather than full latent variable SEM
2. **Transparent terminology** for the network analysis component, avoiding any potential confusion with Bayesian probabilistic modelling
3. **Robust methodological justification** supported by recent literature on small-sample mediation analysis
4. **Comprehensive replication materials** enabling full reproducibility of our findings

We believe these changes strengthen the manuscript substantially and we are grateful for the opportunity to improve our work through this review process. *We remain at the Reviewer’s disposal should any further clarification be required.*

# Reviewer report: Reviewer response for version 1

**DOI:** 10.21956/openreseurope.21967.r61002

**Published:** 2025-10-10

**Authors:** Javier Barbero

**Affiliations:** 1 Department of Economics, Universidad Autonoma de Madrid Facultad de Ciencias Economicas y Empresariales (Ringgold ID: 88092), Madrid, Community of Madrid, Spain

The manuscript uses data from the World Values Survey to analyse the influence of social attitudes on the quality of institutions and development. Social attitudes are measured through the replies to three questions from the survey on gender equality, environmental migration, and the impact of immigration on development. Then, attitude groups are formed by combining the replies, and three main clusters are presented as positive, neutral, and negative groups. The manuscript concludes by providing some policy recommendations and avenues for future research.

 Most of the manuscript is easy to read and follow for a general audience. However, section 4 on the methodology is quite technical and could be hard to follow for readers not familiar with it. A more intuitive explanation of the methodology will help readers understand why these methodologies were chosen and how they work. Readers with a background in economics, social sciences, and policy studies will benefit from such an intuitive explanation.

 The authors provide replication files. Not all source data is available, as the authors do not have the right to redistribute it. However, the authors clearly explain how to replicate the results if the reader can get the source data following the instructions provided.

If applicable, is the statistical analysis and its interpretation appropriate?

Yes

Is the study design appropriate and is the work technically sound?

Yes

Is the work clearly and accurately presented and does it engage with the current literature?

Yes

Are the conclusions drawn adequately supported by the results?

Yes

Are sufficient details of methods and analysis provided to allow replication by others?

Partly

Are all the source data and materials underlying the results available?

Partly

Reviewer Expertise:

My research area is Economics. I can only assess whether the manuscript is written in scientific form and follows the scientific method. I do not know the methodology used, and I cannot assess whether its use was appropriate. Another reviewer, an expert in Bayesian Network Analysis (BNA) and Structural Equation Modeling (SEM), should assess section 4.

I confirm that I have read this submission and believe that I have an appropriate level of expertise to confirm that it is of an acceptable scientific standard.
